# One-pass-throw-away learning for cybersecurity in streaming non-stationary environments by dynamic stratum network

**DOI:** 10.1371/journal.pone.0202937

**Published:** 2018-09-06

**Authors:** Mongkhon Thakong, Suphakant Phimoltares, Saichon Jaiyen, Chidchanok Lursinsap

**Affiliations:** 1 Department of Mathematics and Computer Science, Faculty of Science, Chulalongkorn University, Bangkok, Thailand; 2 Department of Computer Science, Faculty of Science, King Mongkut’s Institute of Technology Ladkrabang, Bangkok, Thailand; Victoria University, AUSTRALIA

## Abstract

Throughout recent times, cybersecurity problems have occurred in various business applications. Although previous researchers proposed to cope with the occurrence of cybersecurity issues, their methods repeatedly replicated the training processes for several times to classify datasets of these problems in streaming non-stationary environments. In dynamic environments, the conventional methods possibly deteriorate the adaptive solution to prevent these issues. This research proposes a one-pass-throw-away learning using the dynamical structure of the network to solve these problems in dynamic environments. Furthermore, to speed up the computational time and to maintain a minimum space complexity for streaming data, the new concepts of learning in forms of recursive functions were introduced. The information gain-based feature selection was also applied to reduce the learning time during the training process. The experimental results signified that the proposed algorithm outperformed the others in incremental-like and online ensemble learning algorithms in terms of classification accuracy, space complexity, and computational time.

## Introduction

The assumption of current machine learning systems is designed to handle stationary data in which training and testing data could be independently distributed. This assumption is often violated in the study of cybersecurity problems, as the systems typically operate with a non-stationary environment called concept drift [1]. However, if machine learning systems are deployed online, an attacker may attempt to seek their vulnerabilities and evade the predicted performances by manipulating data on a variety of scenarios (situations) [2, 3]. For the Internet of Things (IoT) security, risks and threats may occur on devices connected to the Internet in uncertain situations [4, 5]. Moreover, cloud computing systems can be threatened from intruders in terms of the vulnerability of data integrity, authentication, and denial of service [6, 7]. Therefore, recent security issues of machine learning systems led to complications in several real-life applications such as network traffic analysis, fraud detection, spam filtering, intrusion detection, phishing detection, ransomware detection, and malware classification [8–13]. As the number of attacks and its severity are expected to continuously increase over the next several years, the main concern of these cyber applications is how the system can be protected from such attacks.

The learning of information acquired from cybersecurity domain has been applied for recognizing a data-driven solution as a consequence of a large amount of raw data available and the worldly cyber-attacks made throughout the world [14]. This situation is difficult to overcome with human expertise on these attacks occurring in a variety of scenarios. Learning processes have been developed by merging knowledge learned from previously seen data, along with an analysis of human expertise, to provide a scalable solution. The adaptive solution of learning has been widely designed in several security applications. For example, supervised learning techniques of classification are used for spam filtering [10, 15, 16]. Alternatively, graph-based learning can also be applied to find relationships between the reviews and their corresponding authors [17]. In addition, various techniques of machine learning, i.e. decision tree algorithm [18], ensemble and hybrid classifiers [19], support vector machines [20], bayesian network [21], and genetic algorithm [22], were feasibly applied to enhance the intrusion detection systems. Furthermore, many statistical detection techniques [21] were used for anomaly detection in network traffic. Hybrid approaches combining supervised and unsupervised techniques based on machine learning algorithms were also used for the detection of network attacks [23]. Other techniques introduced self-structuring neural network [24] or associative classification [25] for predicting the phishing website.

With the characteristic of a non-stationary environment, the changes in data classes can conduce to the structure of dynamic data distribution over a period of time. Types of changes in non-stationary environments concern gradual changes, recurrent concepts, and sudden drift [26, 27]. As summarized in [1], the learning of non-stationary environments was introduced. Recently, learning methods have been mainly aiming to solve several clustering and classification problems. For instance, the streaming ensemble algorithm (SEA) [28] is the first ensemble of classifiers to learn the non-stationary environments for each consecutive windows of time of the training set. Concept drift very fast decision tree (CVFDT) [29] is one of the most well-known streaming data mining methods to cope with concept drift by using a fixed-size window of instances. Ensemble learning under non-stationary environments was proposed to use weighted majority vote (WMV) [30] based on the loss function for analyzing the probability of multiple expert systems. The dynamic weighted majority [31] was proposed to learn under online situations by adding or removing the number of classifiers for tracking concept drift. The arrangements of processing examples described in [32], chunk-based and online ensembles were intended for applications with strict time and memory constraints. The accuracy updated ensemble (AUE) [26] based on chunk-based learning mode was proposed. In this ensemble, all component classifiers were incrementally updated with a chunk of instances. Online learning ensemble, namely the online accuracy updated ensemble (OAUE) [33] was introduced for improving AUE in the aspect of classification and training time. The anticipative dynamic adaptation to concept changes (ADACC) [34] ensemble was proposed to optimize control over the online classifiers by recognizing concepts in incoming instances. Adaptive random forest (ARF) [35], introduced by Gomes et al., was used in the classification of evolving data streams. The learning of changes in the environments considers the purpose to preserve all acquired knowledge. This is accomplished by aggregating new knowledge and retaining existing knowledge, as called *stability-plasticity dilemma* [27].

Although there have been several learning models proposed to deal with the problems of cybersecurity, other issues still remain. The process of several iterations by using ensemble and hybrid classifiers and particularly storing training data in a sliding-window size has been studied to enhance the predicted performance [18–20]. For a streaming non-stationary environment, these models cannot be appropriately applied due to the changes of context over time. The learning of the cybersecurity problems under streaming non-stationary environments must adapt to the structures of the network corresponding to these environments to maintain its performance. An example of occurring changes is found in spam filtering, in which a user has changed his/her interested issues over a period of time as discussed in [16].

To overcome the cybersecurity problems in a streaming non-stationary environment, a new *dynamic stratum network* aiming to provide an adaptive structure, is composed of versatile elliptic function, recursive functions for adjusting the parameters of the network, and an expandable and shrinkable adaptive network to capture the characteristics of available data. The proposed learning algorithm can learn data without retaining all learned data known beforehand, as called *one-pass-throw-away* learning. Those already learned data are removed from the learning process forever. To reduce memory space and computational time, feature selection based on information gain is also applied in this study.

The remainder of this paper is organized as follows. Section 2 briefly summarizes the relevant theoretical background. Section 3 describes the concept of proposed method and the experimental results are shown in Section 4. Section 5 concludes the study.

## Relevant background

This section provides some backgrounds related to the studied problem and the proposed algorithm. The proposed algorithm adapted some partial concepts of one-pass-throw-away to create and to expand hidden neurons capturing data in the learning process. The summary of one-pass-throw-away learning in [36, 37] is the following.

The output of the neuron *k* with respect to an input **x** is computed from a rotated elliptic function as shown in Eq (1).

$$\begin{array}{c} \psi_{k}\left(x\right)=\sum_{i=1}^{d}\frac{{\left({\left(x-C_{k}\right)}^{T}u_{i}^{k}\right)}^{2}}{{\left(w_{i}^{k}\right)}^{2}}-1 \end{array}$$ (1)

where $C_{k}={\left[c_{1}^{k}\;c_{2}^{k}\;...\;c_{d}^{k}\right]}^{T}$ is a center vector of the ellipse represented by a hidden neuron Ω_*k*_, $U_{k}=\left[u_{1}^{k}\;u_{2}^{k}\;...\;u_{d}^{k}\right]$ is a column matrix of data covariance distribution captured by Ω_*k*_. *N*_*k*_ is the number of data captured by Ω_*k*_. $W_{k}={\left[w_{1}^{k}\;w_{2}^{k}\;...\;w_{d}^{k}\right]}^{T}$ is a width vector of Ω_*k*_. *l*_*k*_ is the class label of Ω_*k*_. In other words, each hidden neuron Ω_*k*_ can be denoted by {**C**_*k*_, **U**_*k*_, *N*_*k*_, **W**_*k*_, *l*_*k*_}.

Any two hidden neurons Ω_*a*_ = {**C**_*a*_, **U**_*a*_, *N*_*a*_, **W**_*a*_, *l*_*a*_} and Ω_*b*_ = {**C**_*b*_, **U**_*b*_, *N*_*b*_, **W**_*b*_, *l*_*b*_} can be combined into one new hidden neuron Ω_*c*_ with a particular condition. After combining, the new parameters are computed as follows.

$$\begin{array}{c} C_{c}^{=}\frac{1}{N_{a}+N_{b}}(N_{a}C_{a}+N_{b}C_{b}) \end{array}$$ (2)

$$\begin{array}{c} U_{c}=\frac{N_{a}}{N_{a}+N_{b}}U_{a}+\frac{N_{b}}{N_{a}+N_{b}}U_{b}+\frac{N_{a}N_{b}}{{\left(N_{a}+N_{b}\right)}^{2}}\times \left(C_{a}-C_{b}\right){\left(C_{a}-C_{b}\right)}^{T} \end{array}$$ (3)

$$\begin{array}{c} N_{c}=N_{a}+N_{b} \end{array}$$ (4)

$$\begin{array}{c} w_{i}^{\left(new\right)}=\sqrt{2\pi |\lambda_{i}|};\;\;\;\;1\leq i\leq d \end{array}$$ (5)

where λ_*i*_ is the *i*^*th*^ eigenvalue of the covariance matrix **U**_*c*_. After successful combining, Ω_*a*_ and Ω_*b*_ are removed from the network.

The brief of initializing a width vector given in [38] can be described by the following procedure.

**Procedure 1: Initializing the appropriate width vector**

1. Find the maximal and minimal distance values within the training set by using the Euclidean distance.

2. Set the number of bins *B* as twice the number of classes.

3. Compute the length of each bin by using *B* and the gap between the maximal and minimal distance values.

4. Count the training data in each bin based on the distance values.

5. Select a bin containing most of the data.

6. Set the initial width vector as the mid-value of the bin in step 5.

## Proposed method

**Fig 1:**
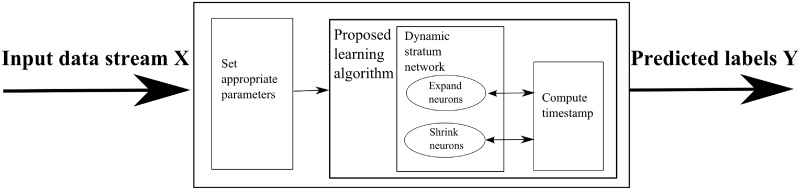
Overview of the dynamic stratum (Dyn-Stratum) model.

This study focuses on one-pass-throw-away learning to maintain less space complexity and computational time in a streaming non-stationary environment of cybersecurity problems. The learning can adjust the structure of network with changing in the environment. Furthermore, if there exists a sub-network in a class *i* whose all data are eventually changed for some unknown reasons, the relevant neurons and their links must be entirely removed. The overview of the proposed method for classifying a streaming non-stationary environment can be shown in Fig 1. This method captures the dynamic network that can adjust the structure according to the incoming data. Meanwhile, the entire data is removed forever after the learning process. These mentioned problems are concentrated in our proposed method as a viable solution with the following issues. The first issue considers the case where dynamic network structure must adjust accordingly to the unexpected situations arriving continuously. Along with the proposed dynamic network structure, the second issue is the dimensional reduction of features to speed up the training process. The details of each issue are described in the following sections.

### Dynamical structure of proposed network

**Fig 2:**
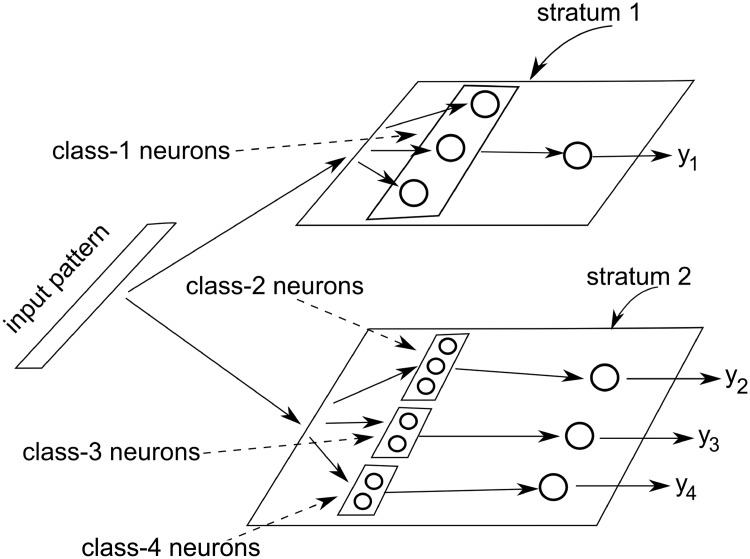
Our proposed structure of the network for handling cybersecurity problems.

The proposed Dynamic Stratum (Dyn-Stratum) network is developed as a streaming incremental framework for handling concept drift in cybersecurity problems. There are three layers in the Dyn-Stratum network. The first layer is the input layer consisting of a set of neurons whose size is equal to the dimensions (attributes) of the input vector. The second layer (hidden layer) connected to the first layer consists of a set of neurons in each class. The sub-network of this layer is composed of two strata with timestamp of the streaming data in the training process. The first stratum contains neurons created and expanded with the most recent timestamp. The second stratum contains neurons which are moved and shrunk from the first stratum corresponding to the training period with the less timestamp. The third layer is the output layer, which consists of a set of output neurons corresponding to the number of classes. The proposed Dyn-Stratum network can be shown in Fig 2. Assuming that there are four classes which are 1, 2, 3, and 4, without loss of generality, the network of each class which has only one output denoted by *y*_*i*_ can be defined. All classes in each stratum are learned by a 3-layer feed-forward network. The hidden layer of this network includes many groups of neurons, i.e. one group for one class.

### Proposed learning algorithm

**Fig 3:**
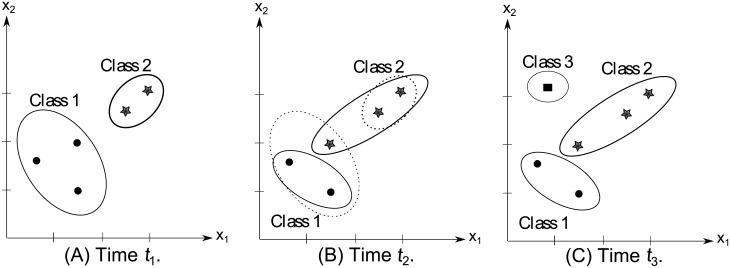
An example of how Dyn-Stratum works.

To clarify the proposed Dyn-Stratum concept, an example of how the proposed learning algorithm works is shown in Fig 3. At the beginning time *t*_1_, assumes that there are two neurons of Class 1 and Class 2 denoted by thick dots and stars, respectively. There are three data within the neuron of Class 1 and two data within the neuron of Class 2 with the recent timestamp. All classes are stored in the stratum 1. At the second time *t*_2_, the label of a datum in Class 1 changed to Class 2. This causes the neuron of Class 1 to shrink and to assign in the stratum 2 corresponding to the less timestamp, meanwhile the neuron of Class 2 expanded due to the class label of data in Class 1 stored in the stratum 1, which has been changed. At the last time *t*_3_, one new datum denoted by the square is captured by the neuron of Class 3 stored in the stratum 1, but none of the others changed in Class 1 and Class 2. The three main steps in our proposed algorithm will be concentrated by the following procedure.

**Procedure 2: Learning algorithm**

1. Setting up the appropriate parameters for creating a new neuron.

2. Updating the structure of network with expanding and shrinking neurons.

3. Computing the timestamp of the neuron.

The first step is to define the appropriate parameters for creating a hidden neuron in which these parameters will influence the computational time and classification accuracy of the learning process. Especially, in terms of a width vector, if it is initially computed with appropriate values, the structure of network will also be efficiently updated. The suitable width vector was calculated from the distances among some training data points, as introduced in [38]. The method to compute initial width vector is given in the above-mentioned section.

The second step is to adjust the proposed Dyn-Straum network which can expand and shrink according to the incoming data or class-changed data. When adding an incoming datum, the center vector and the covariance matrix are recursively computed as demonstrated in the following: For any hidden neuron Ω_*k*_, the following parameters {**C**_*k*_, **U**_*k*_, *N*_*k*_, **W**_*k*_, *l*_*k*_} are already learned. Suppose an incoming datum **x**_*j*_ arrives and falls inside the boundary of Ω_*k*_. The value of *ψ*_*k*_ in Eq (1) must be less than or equal to 0. The new center $C_{k}^{\left(new\right)}$ of Ω_*k*_ is computed from the old center $C_{k}^{\left(old\right)}$ by the following recursive function.

$$\begin{array}{c} C_{k}^{\left(new\right)}=\frac{N_{k}}{N_{k}+1}C_{k}^{\left(old\right)}+\frac{x_{j}}{N_{k}+1} \end{array}$$ (6)

The new covariance matrix $U_{k}^{\left(new\right)}$ of Ω_*k*_ is recursively computed from the old covariance matrix $U_{k}^{\left(old\right)}$ and the new center $C_{k}^{\left(new\right)}$ as follows.

$$\begin{array}{c} U_{k}^{\left(new\right)}=\frac{N_{k}}{N_{k}+1}(U_{k}^{\left(old\right)}+C_{k}^{\left(old\right)}C{{}_{k}^{\left(old\right)}}^{T})+\frac{x_{j}{x_{j}}^{T}}{N_{k}+1}-C_{k}^{\left(new\right)}C{{}_{k}^{\left(new\right)}}^{T} \end{array}$$ (7)

The total data captured by Ω_*k*_ becomes *N*_*k*_ + 1. The detail of process can be conducted in the *ExpandingNeuron* function.

However, in the case of removing a datum from a hidden neuron Ω_*k*_, the following equations can be computed to acquire the center vector and the covariance matrix as follows.

$$\begin{array}{c} C_{k}^{\left(new\right)}=\frac{N_{k}}{N_{k}-1}C_{k}^{\left(old\right)}-\frac{x_{j}}{N_{k}-1} \end{array}$$ (8)

$$\begin{array}{c} U_{k}^{\left(new\right)}=\frac{N_{k}}{N_{k}-1}(U_{k}^{\left(old\right)}+C_{k}^{\left(old\right)}C{{}_{k}^{\left(old\right)}}^{T})-\frac{x_{j}{x_{j}}^{T}}{N_{k}-1}-C_{k}^{\left(new\right)}C{{}_{k}^{\left(new\right)}}^{T},\;\text{where}\;N_{k}>1. \end{array}$$ (9)

The remaining data captured by Ω_*k*_ becomes *N*_*k*_ − 1. However, if there is no data captured by Ω_*k*_, its links must be entirely removed. The detail of process can be conducted in the *ShrinkingNeuron* function.

The third step is to compute the timestamp of Ω_*k*_ by using the number of data captured by Ω_*k*_ and previous learning time. The value of timestamp *t*_*k*_ of the neuron Ω_*k*_ can be computed recursively by:

$$\begin{array}{c} t_{k}^{\left(new\right)}=t_{k}-log\frac{N_{k}}{N_{k}^{\left(new\right)}} \end{array}$$ (10)

where *N*_*k*_, $N_{k}^{\left(new\right)}$ are the present number and the updated number of data captured by Ω_*k*_, respectively. Furthermore, the testing step is to predict the class of any testing datum **x**_*j*_ denoted as *y*(**x**_*j*_). Let **H** be a set of hidden neurons in two strata.

$$\begin{array}{c} y\left(x_{j}\right)=\underset{k}{\arg\max}(t_{k}|\Omega_{k}\in H\;and\;\psi_{k}\left(x_{j}\right)\leq 0) \end{array}$$ (11)

or, when **x**_*j*_ is outside all neurons,

$$\begin{array}{c} y\left(x_{j}\right)=\underset{k}{\arg\min}(\;\psi_{k}\left(x_{j}\right)|\Omega_{k}\in H) \end{array}$$ (12)

when *ψ*_*k*_(**x**_*j*_) according to Eq (1) is the output of a neuron Ω_*k*_ in any stratum.

The proposed learning algorithm, as namely *Dyn-Stratum algorithm*, is summarized with three main sub-processes as follows:

1. Creating a new hidden neuron called *CreatingNewNeuron*.
2. Expanding and updating relevant parameters of the hidden neuron called *ExpandingNeuron*.
3. Removing data captured by the hidden neuron called *ShrinkingNeuron*.

**Function 1: CreatingNewNeuron**(**x**_*j*_, *H*, **W**_0_)

1. Compute *H* = *H* + 1.

2. Set the new center vector $C_{H}^{\left(new\right)}=x_{j}$.

3. Set the new covariance matrix of the neuron *H*, i.e. $U_{H}^{\left(new\right)}=0$ (zero matrix).

4. Set the width vector of the neuron *H*, i.e. **W**_*H*_ = **W**_0_.

5. Set the parameter *N*^*H*^ = 1 and *l*^*H*^ as the class of **x**_*j*_.

6. Set the new timestamp as *t*_*H*_ = 1.

7. Return Ω_*H*_ and *t*_*H*_.

**Function 2: ExpandingNeuron**(**x**_*j*_, **C**_*k*_, **U**_*k*_, *N*_*k*_, **W**_*k*_, *l*_*k*_, *t*_*k*_)

1. Set $C_{k}^{\left(old\right)}=C_{k}$, $t_{k}^{\left(old\right)}=t_{k}$, and $U_{{}_{k}}^{\left(old\right)}=U_{k}$.

2. Update the center vector $C_{k}^{\left(new\right)}$ by $C_{k}^{\left(new\right)}=\frac{N_{k}}{N_{k}+1}C_{k}^{\left(old\right)}+\frac{x_{j}}{N_{k}+1}$.

3. Update the covariance matrix $U_{k}^{\left(new\right)}$ by $U_{k}^{\left(new\right)}=\frac{N_{k}}{N_{k}+1}\left(U_{k}^{\left(old\right)}+C_{k}^{\left(old\right)}C_{k}^{\left(old\right)}{}^{{}^{T}}\right)+\frac{x_{j}x_{j}{}^{T}}{N_{k}+1}-C_{k}^{\left(new\right)}C_{k}^{\left(new\right)}{}^{{}^{T}}$.

4. Update the width vector $W_{k}={\left[w_{1}^{k}\;w_{2}^{k}\;...\;w_{d}^{k}\right]}^{T}$ by $w_{i}^{k}=w_{i}^{k}+\sqrt{2\pi |\lambda_{i}|},\;\;1\leq i\leq d$.

5. Compute *N*_*k*_ = *N*_*k*_ + 1.

6. Let *l*_*k*_ be the class of neuron Ω_*k*_.

7. Update the timestamp *t*_*k*_ by $t_{k}=t_{k}^{\left(old\right)}-log\frac{N_{k}}{N_{k}+1}$.

8. Return Ω_*k*_ and *t*_*k*_.

**Function 3: ShrinkingNeuron**(**x**_*j*_, **C**_*k*_, **U**_*k*_, *N*_*k*_, **W**_*k*_, *l*_*k*_, *t*_*k*_)

1. Compute the number of data in Ω_*k*_ by *N*_*tmp*_ = *N*_*k*_ − 1. (if *N*_*tmp*_ = 0, remove Ω_*k*_ and its link from the network).

2. Set $C_{k}^{\left(old\right)}=C_{k}$, $t_{k}^{\left(old\right)}=t_{k}$, and $U_{{}_{k}}^{\left(old\right)}=U_{k}$.

3. Update the center vector $C_{k}^{\left(new\right)}$ by $C_{k}^{\left(new\right)}=\frac{N_{k}}{N_{k}-1}C_{k}^{\left(old\right)}-\frac{x_{j}}{N_{k}-1}$.

4. Update the covariance matrix $U_{k}^{\left(new\right)}$ by $U_{k}^{\left(new\right)}=\frac{N_{k}}{N_{k}-1}\left(U_{k}^{\left(old\right)}+C_{k}^{\left(old\right)}C_{k}^{\left(old\right)}{}^{{}^{T}}\right)-\frac{x_{j}x_{j}{}^{T}}{N_{k}-1}-C_{k}^{\left(new\right)}C_{k}^{\left(new\right)}{}^{{}^{T}}$.

5. Update the width vector $W_{k}={\left[w_{1}^{k}\;w_{2}^{k}\;...\;w_{d}^{k}\right]}^{T}$ by $w_{i}^{k}=\sqrt{2\pi |\lambda_{i}|},\;\;1\leq i\leq d$.

6. Let *l*_*k*_ be the class of neuron Ω_*k*_.

7. Update the timestamp *t*_*k*_ by $t_{k}=t_{k}^{\left(old\right)}-log\frac{N_{k}}{N_{k}-1}$.

8. Return Ω_*k*_ and *t*_*k*_.

**Algorithm 1: Dyn-Stratum**

**Input:** A set of data chunks, $ℑ=\{X_{i}|i=1,2,...,N\}$.

**Output:** The Dyn-Stratum network with *H* hidden neurons and the timestamp *t* in both stratum 1 and stratum 2, **P** = {Ω_*k*_, *t*_*k*_|1 ≤ *k* ≤ *H*}.

1. Let *H*_1_ = 0 and *H*_2_ = 0. (% *H*_1_ and *H*_2_ are the number of neurons in stratum 1 and stratum 2, respectively.

2. Let **W**_0_ be the width vector computed by *Procedure 1*.

3. **While** **X**_*i*_ is not empty **do**

4.  Introduce **x**_*j*_ ∈ **X**_*i*_.

5.  **If** (*H*_1_ ≠ 0) and (*H*_2_ ≠ 0) **then**

6.   Find the closest neuron with same class of **x**_*j*_ in each stratum by using the Mahalanobis distance with,

$$k1=\underset{h}{\arg\min}\{\sqrt{{\left(x_{j}-C_{h}\right)}^{T}{U_{h}}^{-1}\left(x_{j}-C_{h}\right)}\;|1\leq h\leq H_{1}\},$$

$$k2=\underset{h}{\arg\min}\{\sqrt{{\left(x_{j}-C_{h}\right)}^{T}{U_{h}}^{-1}\left(x_{j}-C_{h}\right)}\;|1\leq h\leq H_{2}\}$$

where **U**_*h*_^−1^ is defined as the inverse covariance matrix of the *h*^*th*^ neuron.

7.   **If** (*ψ*_*k*1_(**x**_*j*_) ≤ 0) and (*ψ*_*k*2_(**x**_*j*_) ≤ 0) **then**

8.    *ShrinkingNeuron*(**x**_*j*_, **C**_*k*2_, **U**_*k*2_, *N*_*k*2_, **W**_*k*2_, *l*_*k*2_, *t*_*k*2_). % in stratum 2.

9.    *ExpandingNeuron*(**x**_*j*_, **C**_*k*1_, **U**_*k*1_, *N*_*k*1_, **W**_*k*1_, *l*_*k*1_, *t*_*k*1_). % in stratum 1.

10.   **Else If** *ψ*_*k*1_(**x**_*j*_) ≤ 0) and *ψ*_*k*2_(**x**_*j*_) > 0) **then**

11.    *ExpandingNeuron*(**x**_*j*_, **C**_*k*1_, **U**_*k*1_, *N*_*k*1_, **W**_*k*1_, *l*_*k*1_, *t*_*k*1_). % in stratum 1.

12.   **Else If** (*ψ*_*k*1_(**x**_*j*_) > 0) and (*ψ*_*k*2_(**x**_*j*_) ≤ 0) **then**

13.    *ExpandingNeuron*(**x**_*j*_, **C**_*k*2_, **U**_*k*2_, *N*_*k*2_, **W**_*k*2_, *l*_*k*2_, *t*_*k*2_). % in stratum 2.

14.   **Else**

15.    *CreatingNewNeuron*(**x**_*j*_, *H*_1_, **W**_0_). % in stratum 1.

16.   **EndIf**

17.  **Else If** (*H*_1_ ≠ 0) **then**

18.   Find the closest neuron of **x**_*j*_ in stratum 1 by,

$$k1=\underset{h}{\arg\min}\{\sqrt{{\left(x_{j}-C_{h}\right)}^{T}{U_{h}}^{-1}\left(x_{j}-C_{h}\right)}\;|1\leq h\leq H_{1}\}.$$

19.   **If** *class*(**x**_*j*_) ≠ the class of neuron Ω_*k*1_ **then**

20.    *CreatingNewNeuron*(**x**_*j*_, *H*_1_, **W**_0_). % in stratum 1.

21.    Move the neuron Ω_*k*1_ into stratum 2.

22.   **Else**

23.    *ExpandingNeuron*(**x**_*j*_, **C**_*k*1_, **U**_*k*1_, *N*_*k*1_, **W**_*k*1_, *l*_*k*1_, *t*_*k*1_).

24.   **EndIf**

25.  **Else If** (*H*_2_ ≠ 0) **then**

26.   Find the closest neuron of **x**_*j*_ in stratum 2 by,

$$k2=\underset{h}{\arg\min}\{\sqrt{{\left(x_{j}-C_{h}\right)}^{T}{U_{h}}^{-1}\left(x_{j}-C_{h}\right)}\;|1\leq h\leq H_{2}\}.$$

27.   **If** *class*(**x**_*j*_) ≠ the class of neuron Ω_*k*2_ **then**

28.    *CreatingNewNeuron*(**x**_*j*_, *H*_2_, **W**_0_). % in stratum 2.

29.    Move the neuron Ω_*k*2_ into stratum 1.

30.   **Else**

31.    *ExpandingNeuron*(**x**_*j*_, **C**_*k*2_, **U**_*k*2_, *N*_*k*2_, **W**_*k*2_, *l*_*k*2_, *t*_*k*2_).

32.   **EndIf**

33.  **Else**

34.   *CreatingNewNeuron*(**x**_*j*_, ***H***_1_, **W**_0_). % in stratum 1.

35.  **EndIf**

36.  **If** there exist Ω_*a*_ and Ω_*b*_ neurons overlapping of the same class in any stratum **then**

37.   Combine Ω_*a*_ and Ω_*b*_ neurons into new neuron Ω_*c*_ with Eqs 2–5.

38.  **X**_*i*_ = **X**_*i*_ − {**x**_*j*_}

39. **EndWhile**

### Ranking relevant attributes

As the aforementioned proposed learning algorithm, the main processes of learning depend on the computations of the center vector and the covariance matrix (also known as dispersion matrix). If the process, in terms of the high number of features (attributes) is computed, this will consume most of the computational learning time. Although the features may contain a large number of characteristics, not all of them are essential. Some features are redundant or irrelevant. Redundant features are highly correlated to other features and do not have additional information to the target learning task whilst irrelevant features do not have any helpful information with regards to the context [39, 40]. Thus, the main objective of ranking the most important features is to enhance the classification accuracy and to correctly represent the characteristics of patterns. An example introduced in [40], demonstrates the grouping and clustering of alerts for detecting attacks by using the similarity of features.

Attribute ranking is a filter method of feature selection. Because of its simplicity, the method is successively used for practical applications. The attribute ranking method is implemented by applying Information Gain (IG) before classification to filter out the less relevant attributes [39]. The Information Gain is frequently used as a term-goodness criterion in different applications of classification problems. The proposed algorithm for ranking relevant attributes is conducted from information entropy to compute Information Gain and return the sort order of most useful attributes (highest Information Gain) to the lowest. The process of ranking attributes can be detailed with the following algorithm.

**Algorithm 2: Ranking Attributes based on Information Gain**

**Input:** Training set **X** with the set of class labels **Y**.

**Output:** *k* attributes.

1. Let *D* be the number of attributes of **X**.

2. **For** each *y*_*i*_ ∈ **Y** **do**

3.  Compute *P*(*y*_*i*_). % probability of class *y*_*i*_.

4. **EndFor**

5. $Entropy\left(Y\right)=-\sum_{y_{i}\in Y}P\left(y_{i}\right){\text{log}}_{2}\left(P\left(y_{i}\right)\right)$.

6. Set *d* = 1.

7. **While** *d* ≤ *D* **do**

8.  Compute *P*(**Y**|**X**_*d*_).

9.  ϒ_*d*_ = *P*(**Y**|**X**_*d*_) * log_2_ *P*(**Y**|**X**_*d*_).

10.  *G*_*d*_ = *Entropy*(**Y**) − ϒ_*d*_. % computing weight of attribute *d*.

11.  *d* = *d* + 1.

12. **EndWhile**

13. Sort the set of weight attributes {*G*_*d*_; 1 ≤ *d* ≤ *D*} in a descending order.

14. Select the best *k* attributes.

## Experiments

The benchmarked datasets were collected from three real-world concept drift datasets in cybersecurity studies, as shown in Table 1. These datasets consist of a large number of dimensional attributes and are popular as they have experimented in many machine learning literatures. The Spam dataset taken from [41] represents the task of separating malicious spam emails from legitimate ones. Phishing websites dataset [42] was collected from malicious web pages. The Phishing website is one of the many worldwide challenging security problems. The NLS-KDD dataset [43] was obtained from the application of intrusion detection systems, where the main focus is on filtering malicious network traffic. All attributes in the datasets are converted to numeric type before applying the proposed algorithms. These datasets depict the concept drift, but the type of drifts cannot be determined in advance. The description of the datasets is detailed in the next subsection.

**Table 1:** The benchmarked datasets on real-world cybersecurity problem.

Dataset	# Instances	# Attributes	# Classes
Spam	9324	499	2
Phishing	11055	30	2
NLS-KDD	148517	41	5

### Dataset description

Three real-world concept drift datasets were chosen from the domain of cybersecurity, as summarized in Table 1, the datasets consist of the number of instances, the number of features (attributes), and the number of class labels. The Spam dataset contains 9,324 instances and was constructed from the email messages of the Spam Assassin Collection. Of all the instances, there are 500 attributes including class labels, such that each attribute stands for the occurrence of a single word in an instance (e-mail). As previously mentioned in [41], this dataset contains spam message characteristics which will gradually change over time.

The Phishing websites dataset was acquired from the UCI Irvine machine learning repository [42]. There are 11,055 website samples, such that each sample consists of 30 attributes. The 6,157 legitimate websites are defined to a class label of “+1”, while the 4,898 phishing websites are defined to a class label of “-1”. All of them were collected from PhishTank archive, MillerSmiles archive, and Google’s searching operator.

The NSL-KDD dataset is a modified version of the KDD Cup 99 data set, which is studied as the benchmarked dataset of cybersecurity problems. This dataset includes TCP connection records that consist of 41 informational attributes and one labelling attribute classified into one of four types of attacks or normal connection. Among the 41 attributes, there are 32 continuous attributes and 9 nominal attributes. In addition, for further evaluation, this experiment transformed the dataset into a two-class problem consisting of abnormal and normal classes as well.

### Performance evaluation

This study concerns the evaluation, in terms of performance, for the proposed Dyn-Stratum algorithm when compared to several other existing classification algorithms; including incremental learning for non-stationary environments (Learn++.NSE) [27], weighted majority vote (WMV) [30], Anticipative Dynamic Adaptation to Concept Drift (ADACC) [34], and Adaptive Random Forests (ARF) [35]. These compared algorithms were implemented with chunk-based and online learning modes for non-stationary streams. Chunk-based mode processes incoming data in chunks, where each chunk contains a fixed number of training instances. Online mode learns each incoming datum separately, rather than in chunks, and then discards it. The evaluation of the performance of proposed Dyn-Stratum, Learn++.NSE, WMV, ADACC, and ARF methods were implemented with MATLAB and Massive Online Analysis (MOA) framework [44]. All experiments could be evaluated with two settings. The first evaluation comprises space and time complexities as well as overall classification (e.g., accuracy, precision, recall, f-measure, and geometric mean) in details of the equations as will be given afterward. The measures are evaluated on the whole data stream based on 5-fold cross-validation technique. In cross-validation, the whole data set was sequentially divided into five subsets of instances. In each iteration, the four subsets were used as for training to derive a method and then the rest was used to test the method. The process was accomplished five times repeatedly. In addition, to evaluate other important measures, this study employs true positive (TP), false negative (FN), false positive (FP), and true negative (TN) called confusion matrix, as shown in Table 2. The confusion matrix was used to explain the calculation such as *Precision*, *Recall*, *F-measure*, and *Geometric mean (G-mean)* with the following equations.

$$\begin{array}{c} Precision=\frac{TP}{TP+FP} \end{array}$$ (13)

$$\begin{array}{c} Recall=\frac{TP}{TP+FN} \end{array}$$ (14)

$$\begin{array}{c} F-measure=\frac{2*Recall*Precision}{Recall+Precision} \end{array}$$ (15)

$$\begin{array}{c} G-mean=\sqrt{\frac{TP}{TP+FN}*\frac{TN}{TN+FP}} \end{array}$$ (16)

**Table 2:** Confusion matrix for a binary classification.

Actual class	Predicted class	
	Yes	No
Yes	TP	FN
No	FP	TN

In this setting, to accurately evaluate the important attributes based on Information Gain, as introduced by Algorithm 2 in an earlier section, the comparison results were also reported in our experiments.

The second evaluation is an incremental learning curve of the benchmarked datasets separated into chunks using test-then-train strategy with respect to the classification accuracy of each algorithm.

The experimental set-up was conducted to fairly evaluate the performance of algorithms. The training data were randomly partitioned into several chunks to test our concept of one-pass-throw-away learning. For our experimental set-up, the same set of data used by those compared algorithms was also used in our experiment. The initial width value calculated in *Procedure 1* was used for setting the appropriate width vector of the proposed Dyn-Stratum algorithm. As introduced in [27], the sigmoid parameters of the Learn++.NSE were equal to 0.5 and 1.0. The classifiers were set as classification and regression (CART) both the Learn++.NSE and WMV methods. On the other hand, Naive Bayes classifier was used as base learners of ADACC method since the method usually learns incrementally and is frequently employed in online learning mode. The parameters of threshold and ensemble size in ARF method were set according to the condition, as previously mentioned in [35].

### Experimental results by using cross-validation strategy

In this section, the evaluation of the performance is designed for each algorithm by using cross-validation technique. The percentage of the average accuracy with standard deviation, the number of neurons, and the training time are shown in Tables 3–6. Note that other existing methods do not define the neurons as the structure of the network. The word neuron is used to represent the number of classifiers (or trees) of all compared methods. Table 3 shows the comparison results on the Spam dataset. The comparison results from different methods for Phishing and NSL-KDD datasets are shown in Tables 4 and 5. The result of NSL-KDD dataset transformed into binary classification is also shown in Table 6. Moreover, in terms of ranked attributes based on Information Gain, the average accuracy of the Dyn-Stratum was also maintained to boost the performance for all benchmarked cybersecurity datasets.

**Table 3:** The performance on the *Spam* dataset with two classes over 10 runs.

Method	With all attributes (499 attributes)			With ranked attributes (341 attributes)		
	average accuracy (%) with its standard deviation	# average neurons	average training time (seconds)	average accuracy (%) with its standard deviation	# average neurons	average training time (seconds)
Dyn-Stratum	**95.23(2)**	**3**	627.02	**94.52(2)**	**3**	205.81
Learn++.NSE [27]	77.34(20)	14	198.32	75.47(5)	14	144.14
WMV [30]	85.08(5)	14	**45.16**	84.45(7)	13	**44.09**
ADACC [34]	94.56(3)	20	698.26	94.13(2)	20	612.25
ARF [35]	85.67(7)	25	169.91	85.46(4)	25	126.19

**Table 4:** The performance on the *Phishing* dataset with two classes over 10 runs.

Method	With all attributes (30 attributes)			With ranked attributes (20 attributes)		
	average accuracy (%) with its standard deviation	# average neurons	average training time (seconds)	average accuracy (%) with its standard deviation	# average neurons	average training time (seconds)
Dyn-Stratum	**92.41(2)**	**4**	**27.21**	**92.18(2)**	**3**	**24.22**
Learn++.NSE	92.21(2)	88	63.68	91.41(3)	88	63.31
WMV	91.18(2)	25	29.73	91.22(2)	25	28.71
ADACC	90.95(2)	20	339.85	90.12(12)	20	329.21
ARF	88.38(4)	25	49.01	87.58(5)	25	43.28

**Table 5:** The performance on the *NSL-KDD* dataset with four attacks and normal class over 10 runs.

Method	With all attributes (41 attributes)			With ranked attributes (26 attributes)		
	average accuracy (%) with its standard deviation	# average neurons	average training time (seconds)	average accuracy (%) with its standard deviation	# average neurons	average training time (seconds)
Dyn-Stratum	**91.23(2)**	**7**	567.14	**91.41(2)**	**12**	650.73
Learn++.NSE	87.58(7)	79	7730.82	87.48(5)	75	7528.94
WMV	91.21(3)	15	**498.61**	91.20(3)	15	**430.78**
ADACC	88.04(6)	20	6039.27	85.07(5)	20	5748.51
ARF	89.34(5)	20	4802.15	84.28(5)	20	4874.39

**Table 6:** The performance on the modified *NSL-KDD* dataset with two classes over 10 runs.

Method	With all attributes (41 attributes)			With ranked attributes (26 attributes)		
	average accuracy (%) with its standard deviation	# average neurons	average training time (seconds)	average accuracy (%) with its standard deviation	# average neurons	average training time (seconds)
Dyn-Stratum	**93.78(2)**	**5**	478.85	**94.61(3)**	**8**	532.81
Learn++.NSE	89.14(2)	75	7159.11	88.20(4)	50	3228.18
WMV	92.85(3)	20	**428.67**	92.13(4)	25	**507.79**
ADACC	88.45(2)	20	4145.03	87.39(1)	20	3320.48
ARF	89.23(4)	20	2857.72	88.35(2)	20	2731.64

To evaluate the performance of algorithms based on confusion matrix, G-mean was used to report with the comparison results for cybersecurity datasets in terms of the binary classification. In addition, both G-means with and without process of ranking attributes were also compared to report these experimental results. G-mean of all benchmarked cybersecurity datasets obtained from each algorithm is shown in Table 7. G-mean of our Dyn-Stratum achieved the highest values for experimental results of all benchmarked datasets. Additional performance measures, i.e. precision, recall, and F-measure, are evaluated for all benchmarked cybersecurity datasets, as shown in Tables 8–10. From these results, they are notable that for almost all benchmarked datasets, the Dyn-Stratum gained the highest values.

**Table 7:** *Geometric mean* of the proposed Dyn-Stratum, Learn++.NSE, WMV, ADACC, and ARF methods on cybersecurity datasets.

Method	Spam		Phishing		Modified NSL-KDD with two classes	
	with all attributes (499 attributes)	with ranked attributes (341 attributes)	with all attributes (30 attributes)	with ranked attributes (20 attributes)	with all attributes (41 attributes)	with ranked attributes (26 attributes)
Dyn-Stratum	**0.96**	**0.94**	**0.92**	**0.92**	**0.94**	**0.95**
Learn++.NSE	0.71	0.56	0.90	0.90	0.88	0.87
WMV	0.83	0.54	0.90	0.91	**0.94**	**0.95**
ADACC	0.91	0.91	0.91	0.91	0.89	0.88
ARF	0.79	0.80	0.91	0.91	0.87	0.88

**Table 8:** *Precision* of the proposed Dyn-Stratum, Learn++.NSE, WMV, ADACC, and ARF methods on cybersecurity datasets.

Method	Spam		Phishing		Modified NSL-KDD with two classes	
	with all attributes (499 attributes)	with ranked attributes (341 attributes)	with all attributes (30 attributes)	with ranked attributes (20 attributes)	with all attributes (41 attributes)	with ranked attributes (26 attributes)
Dyn-Stratum	**0.92**	0.89	**0.91**	**0.92**	**0.94**	**0.95**
Learn++.NSE	0.73	0.76	**0.91**	0.91	0.89	0.88
WMV	0.85	0.76	**0.91**	0.91	**0.94**	0.94
ADACC	**0.92**	**0.91**	**0.91**	**0.92**	0.88	0.87
ARF	0.75	0.72	0.74	0.73	0.87	0.86

**Table 9:** *Recall* of the proposed Dyn-Stratum, Learn++.NSE, WMV, ADACC, and ARF methods on cybersecurity datasets.

Method	Spam		Phishing		Modified NSL-KDD with two classes	
	with all attributes (499 attributes)	with ranked attributes (341 attributes)	with all attributes (30 attributes)	with ranked attributes (20 attributes)	with all attributes (41 attributes)	with ranked attributes (26 attributes)
Dyn-Stratum	**0.96**	**0.94**	**0.91**	**0.92**	**0.94**	**0.95**
Learn++.NSE	0.74	0.68	0.90	0.90	0.89	0.88
WMV	0.84	0.65	0.90	0.91	**0.94**	0.94
ADACC	0.92	0.91	**0.91**	0.90	0.88	0.87
ARF	0.70	0.69	0.73	0.69	0.87	0.85

**Table 10:** *F-measure* of the proposed Dyn-Stratum, Learn++.NSE, WMV, ADACC, and ARF methods on cybersecurity datasets.

Method	Spam		Phishing		Modified NSL-KDD with two classes	
	with all attributes (499 attributes)	with ranked attributes (341 attributes)	with all attributes (30 attributes)	with ranked attributes (20 attributes)	with all attributes (41 attributes)	with ranked attributes (26 attributes)
Dyn-Stratum	**0.94**	**0.91**	**0.91**	**0.92**	**0.94**	**0.95**
Learn++.NSE	0.74	0.62	0.90	0.90	0.89	0.88
WMV	0.84	0.61	0.90	0.91	**0.94**	0.94
ADACC	0.92	**0.91**	**0.91**	**0.92**	0.88	0.87
ARF	0.72	0.71	0.74	0.71	0.86	0.85

### Experimental results of streaming data

**Fig 4:**
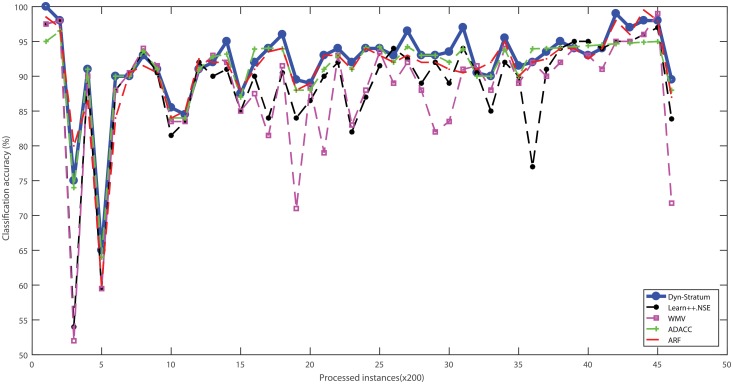
Classification accuracy on the *Spam* dataset.

**Fig 5:**
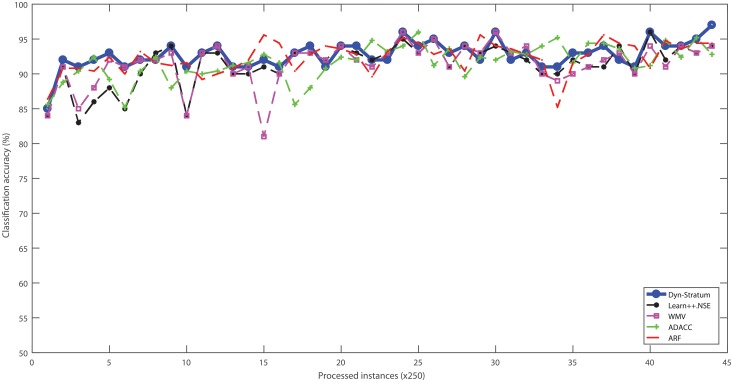
Classification accuracy on the *Phishing* dataset.

**Fig 6:**
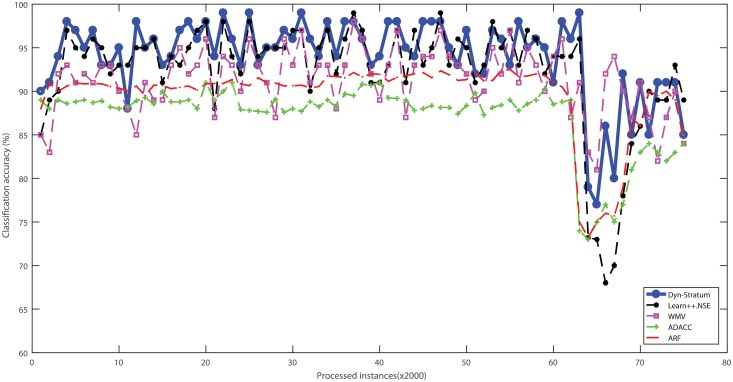
Classification accuracy on the *NSL-KDD* dataset with four attacks and normal class.

**Fig 7:**
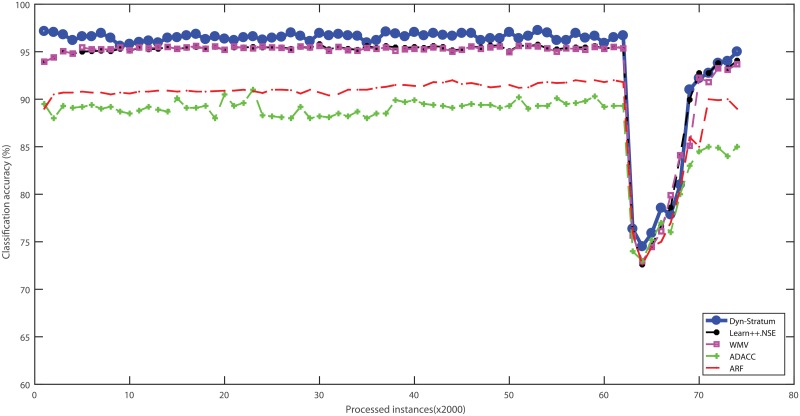
Classification accuracy on the modified *NSL-KDD* dataset with two classes.

Three real-world cybersecurity datasets without ranking attributes were used to evaluate the performance of algorithms with streaming scenarios. These datasets were categorized into data chunks for evaluating the performance based on test-then-train strategy. The percentage of classification accuracy with respect to algorithms of all benchmarked cybersecurity datasets is illustrated in Figs 4–7. For the Spam dataset, the proposed Dyn-Stratum achieved highest classification accuracy in almost all studied instances as shown in Fig 4. For the Phishing dataset, Fig 5 depicts the highest classification accuracy of the Dyn-Stratum algorithm compared to the other algorithms. For the NSL-KDD dataset with four attacks and normal class, Fig 6 shows that the efficient performance of the Dyn-Stratum algorithm is better than the others’. On the other hand, for the NSL-KDD dataset transformed into a two-class problem, the greatest classification accuracy of Dyn-Stratum algorithm is illustrated in Fig 7.

### Discussion

In this study, we focused on the one-pass-throw-away learning for solving the problem of cybersecurity domain in non-stationary scenarios. All experiments were conducted with several data chunks and one-pass-throw-away learning mode. Therefore, the already learned data can be discarded forever after the learning process. The comparison results of experiments on three real-world cybersecurity datasets in non-stationary environments are illustrated in the previous section. The highest average accuracy of the proposed Dyn-Stratum for all benchmarked datasets signified that our Dyn-Stratum network can be flexibly adjusted with the parameters of the network according to consecutive training data. The other methods adjusted the parameters of the network based on the entire dataset. This implies that the consideration of data distribution throughout the space is crucial to speed up the computational time and classification accuracy. For additional measures, the proposed Dyn-Stratum algorithm achieved the highest precision, recall, F-measure, and G-mean compared with the other methods. The computational time of the proposed Dyn-Stratum algorithm is based on the number of times to mainly perform the covariance matrix computation. In the case of test sets with a large dimension of features, the learning process will consume a large amount of learning time. In our experiments, the feature selection based on Information Gain is used to reduce the effect of this case. Consequently, the dimensionality reduction of features to speed up the learning process and the efficiency of classification should be simultaneously considered to improve the performance of method in streaming non-stationary environments.

## Conclusion

The real-world cybersecurity problems in streaming non-stationary environments were studied. A new one-pass-throw-away learning algorithm using the dynamic stratum network named Dyn-Stratum network was proposed to learn these data. The main Dyn-Stratum network comprises two strata. The first stratum designs the dynamical structure based on incrementing a new neuron and expanding the neuron with the recent most timestamp according to incoming data. The second stratum adjusts vigorously to the structure with expanding neurons and removing its connections from the network when the classes of all data have been changed. The center vector and the covariance matrix were also calculated by the recursive functions to reduce the computational time and memory space when data are removed or changed class. The feature selection based on Information Gain was also applied to enhance the performance of computational time. The comparison results between the proposed Dyn-Stratum and the other methods with several benchmarked cybersecurity datasets signified that Dyn-Stratum achieved the minimum structural space complexity, less computational time, and higher classification accuracy than the other methods.
